# DNA metabarcoding for diet analysis and biodiversity: A case study using the endangered Australian sea lion (*Neophoca cinerea*)

**DOI:** 10.1002/ece3.3123

**Published:** 2017-06-12

**Authors:** Tina E. Berry, Sylvia K. Osterrieder, Dáithí C. Murray, Megan L. Coghlan, Anthony J. Richardson, Alicia K. Grealy, Michael Stat, Lars Bejder, Michael Bunce

**Affiliations:** ^1^ Trace and Environmental DNA (TrEnD) Laboratory Department of Environment and Agriculture Curtin University Bentley WA Australia; ^2^ Centre for Marine Science and Technology Curtin University Bentley WA Australia; ^3^ CSIRO Oceans and Atmosphere EcoSciences Precinct Dutton Park Qld Australia; ^4^ Cetacean Research Unit Murdoch University Murdoch WA Australia

**Keywords:** apex predator, dietary scat analysis, DNA metabarcoding, *Neophoca cinerea*, next generation sequencing

## Abstract

The analysis of apex predator diet has the ability to deliver valuable insights into ecosystem health, and the potential impacts a predator might have on commercially relevant species. The Australian sea lion (*Neophoca cinerea*) is an endemic apex predator and one of the world's most endangered pinnipeds. Given that prey availability is vital to the survival of top predators, this study set out to understand what dietary information DNA metabarcoding could yield from 36 sea lion scats collected across 1,500 km of its distribution in southwest Western Australia. A combination of PCR assays were designed to target a variety of potential sea lion prey, including mammals, fish, crustaceans, cephalopods, and birds. Over 1.2 million metabarcodes identified six classes from three phyla, together representing over 80 taxa. The results confirm that the Australian sea lion is a wide‐ranging opportunistic predator that consumes an array of mainly demersal fauna. Further, the important commercial species *Sepioteuthis australis* (southern calamari squid) and *Panulirus cygnus* (western rock lobster) were detected, but were present in <25% of samples. Some of the taxa identified, such as fish, sharks and rays, clarify previous knowledge of sea lion prey, and some, such as eel taxa and two gastropod species, represent new dietary insights. Even with modest sample sizes, a spatial analysis of taxa and operational taxonomic units found within the scat shows significant differences in diet between many of the sample locations and identifies the primary taxa that are driving this variance. This study provides new insights into the diet of this endangered predator and confirms the efficacy of DNA metabarcoding of scat as a noninvasive tool to more broadly define regional biodiversity.

## 1 INTRODUCTION

The majority of marine mammals are generalist predators that consume prey from many trophic levels (Casper, Jarman, Gales, & Hindell, 2007) and therefore potentially influence the community structure of marine environments. As such, the analysis of their diet can provide the opportunity for a comprehensive assessment of the biodiversity present in marine ecosystems (Boyer, Cruickshank, & Wratten, 2015; Casper et al., 2007).

**Figure 1:**
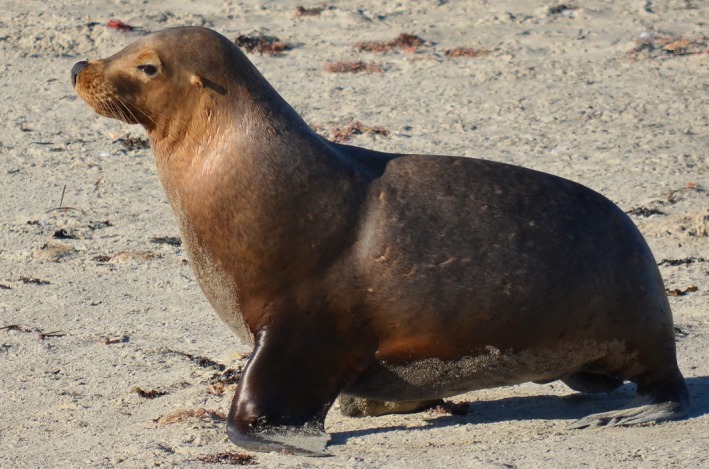
The Australian sea lion (Neophoca cinerea) at Seal Island, Shoalwater Bay, Western Australia

The Australian sea lion (Figure 1) is one of the rarest sea lion species in the world (Hesp et al., 2012) and Australia's only endemic pinniped species (Kirkwood & Goldsworthy, 2013; Ling, 1992). In 2015, there were an estimated 12,290–13,090 individuals remaining in the wild and of these only 16% are found in Western Australia (Goldsworthy, 2015). Australian sea lions are distributed between the Abrolhos Islands in Western Australia and The Pages in South Australia (Ling, 1992), with mostly small and widely scattered colonies, at both remote (Goldsworthy, 2015; Goldsworthy et al., 2009) and near metropolitan areas (Osterrieder, Salgado Kent, & Robinson, 2015, 2016). Despite several dietary studies (Casper et al., 2007; Gales & Cheal, 1992; Kirkwood & Goldsworthy, 2013; Ling, 1992; Peters et al., 2014), much of what this apex predator targets remains poorly defined due to the well‐recognized limits of morphological identification of scat material and/or behavioral studies (Kirkwood & Goldsworthy, 2013). Such reports suggest that the Australian sea lion is a largely nocturnal forager (Kirkwood & Goldsworthy, 2013), although studies of females and pups from Kangaroo Island, South Australia, suggest that their foraging does not follow a diurnal pattern (Costa & Gales, 2003). These previous studies have also shown that sea lions prey mainly on benthic species of fish, sharks, rays, cephalopods, and crustaceans (Kirkwood & Goldsworthy, 2013); however, other evidence also suggests that they prey on rock lobster, swimming crabs, shark eggs, and penguins (McIntosh, Page, & Goldsworthy, 2006). A more recent molecular approach used bacterial cloning and Sanger sequencing of DNA to identify 23 fish and five cephalopod taxa from the scats of 12 female sea lions from two colonies in South Australia (Peters et al., 2014), finding several new taxa upon which sea lions prey.

Observational studies on diet in marine systems can be logistically difficult to conduct and expensive. This is especially true where the animal in question is fast, feeds underwater, and has a large foraging range, as is the case with sea lions (Kirkwood & Goldsworthy, 2013). These problems can be compounded when the study animal is reclusive and/or hunts nocturnally (such as sea lions). In contrast, the collection of sea lion scat is relatively easy as it can be collected by hand from the beaches of known sea lion haul out points. However, morphological analysis of scat has several complications. Firstly, dietary identification relies heavily on the presence of prey remnants, and prey that is relatively undigested may be over represented while highly digested prey may be missed (Boyer et al., 2015; Brown, Jarman, & Symondson, 2012; Shehzad, McCarthy, et al., 2012). Therefore, fleshy or gelatinous targets are unlikely to be detected. In the case of the sea lion*,* smaller cephalopod beaks and fish otoliths digest completely, or are unrecognizable, once they have passed through the digestive tract (Gales & Cheal, 1992; Peters, Ophelkeller, Bott, & Goldsworthy, 2015). This issue is partially attributed to the grinding action of large gastroliths found in the sea lions’ stomach (McIntosh et al., 2006). Gastroliths are large stones that can measure up to approximately 7 cm in diameter and are swallowed by sea lions as ballast (Kirkwood & Goldsworthy, 2013). Secondly, some potential prey species, such as crustaceans, are morphologically similar to one another (Radulovici, Sainte‐Marie, & Dufresne, 2009), making identification of their remains taxonomically challenging. Further, due to the increased rate of survival of cephalopod beaks in comparisons to fish otoliths, reliance on morphological analysis of sea lion scat for dietary analysis can lead to an underestimation of fish but an overestimation of cephalopods consumed (Gales & Cheal, 1992; Peters et al., 2015).

Recent advances in DNA sequencing (and analyzing) environmental samples have enhanced the capacity to identify constituents of fecal material (Pompanon et al., 2012). The use of standard DNA barcodes, PCR, and reference sequence databases facilitates the analysis of prey taxa (or their DNA) that survive in fecal material. DNA metabarcoding approaches (employing next generation sequencing, NGS), where complex mixtures of DNA are extracted and sequenced in parallel, have been successfully applied to several fecal dietary studies with promising results (Berry et al., 2015; Hibert et al., 2013; Murray et al., 2011; Quemere et al., 2013; Shehzad, Riaz, et al., 2012). One of the first studies to exploit DNA metabarcoding, investigated the diet of the Australian fur seal (*Arctocephalus pusillus*; (Deagle, Kirkwood, & Jarman, 2009) and, in a more recent study, the diets of both the Australian (*A. pusillus doriferus*) and long‐nosed fur seals (*A. forsterii*) were compared (Hardy et al., In press). To date, no metabarcoding studies exist to explore the Australian sea lion diet but recently a gut microbiome study was conducted on both wild and captive populations (Delport, Power, Harcourt, Webster, & Tetu, 2016). It is suggested that this type of study could, in future, be combined with a dietary analysis to determine what impact diet has on gut flora.

Using DNA metabarcoding on 36 scat samples, this study seeks to develop and apply multi‐gene metabarcoding assays for the analysis of the diet of the Australian sea lion. The purpose of the results is threefold: (1) to determine the effectiveness of DNA metabarcoding for the dietary analysis of the Australian sea lion and the marine biodiversity that supports them, (2) to assess the predation of commercially valued fishes, and (3) to establish whether this type of study could be used to detect spatial changes in sea lion prey across the southwest of Australia. Importantly, as the Australian sea lion is an endangered species (IUCN Red List; Goldsworthy, 2015), it is of value to develop a holistic picture of what dietary options these apex predators exploit and how these differ spatially and temporally.

## 2 METHODS

### 2.1 Sample collection

**Figure 2:**
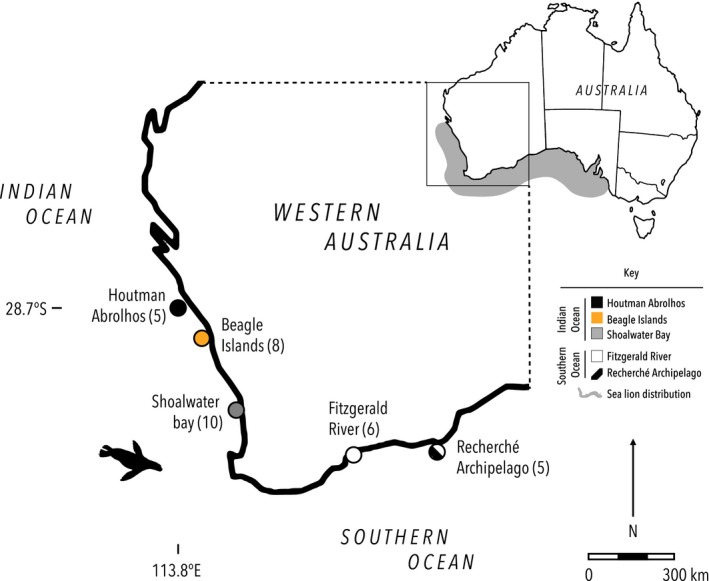
Sampling sites for metabarcoding study; Map of Australia, with inset showing southern Western Australian sampling sites (number of scats in brackets). The shaded areas denote the range of the Australian sea lion across Australia and within Western Australia

In total, 36 fecal samples were collected in sterile containers from islands across five collection sites that stretch 1,500 km of the southwest coast of Western Australia (Figure 2; for greater detail about dates and sites see Table A1). Scat samples were preserved and stored at −20°C.

### 2.2 Metabarcoding assay design

Several PCR assays were designed and/or optimized for use in DNA metabarcoding workflows including the Fish 16S, Ceph 16S, and the Crust 16S assays (Table 1). All primer sets flank hypervariable regions of the 16S rRNA gene and were designed and tested in silico using reference sequences obtained from GenBank. For the Ceph 16S assay, 27 16S sequences from different Western Australian cephalopods were analyzed in silico to identify short conserved areas of the target gene, which will amplify degraded DNA. Similarly, the Crust 16S assay was designed using 13 16S crustacean sequences including crayfish, crab, and prawn species. All newly designed primers were tested against sea lion sequences to ensure no significant amplification of host DNA. To determine the efficacy of the assays, amplifications were optimized on single‐source reference tissue including some crustaceans, a cephalopod, and several species of fish (Table A2).

**Table 1:** Metabarcoding PCR assays and the primer sets used for dietary analysis of *Neophoca cinerea* scat

PCR assay	Primer set used	Target Taxa	Gene	Primer sequence	Amplicon length (bp)	Reference	Assay *T*_m_ (°C)
Bird 12S	12Sa (F)	Birds	12S rRNA	5′ CTGGGATTAGATACCCCACTAT 3′	~230	Cooper (1994)	57
	12Sh (R)			5′ CCTTGACCTGTCTTGTTAGC 3′			
Fish 16S	Fish16sF/D	Fish	16S rRNA	5′ GACCCTATGGAGCTTTAGAC 3′	~200	F‐This study	54
	16s2R (degenerate)			5′ CGCTGTTATCCCTADRGTAACT 3′		R‐Deagle et al. (2007)	
Plank COI	(Plank)Minibar‐Mod‐F	Plankton	COI	5′ TCCACTAATCACAAAGAYATYGGYAC 3′	~127	Berry et al. (2015)	52
	(Plank)Minibar‐Mod‐R			5′ AGAAAATCATAATRAANGCRTGNGC 3′			
Ceph 16S	Ceph16S1_F(deg)	Cephalopods	16S rRNA	5′ GACGAGAAGACCCTADTGAGC 3′	~200	F‐ Peters et al. (2014)	55
	Ceph16SR_Short			5′ CCAACATCGAGGTCGCAATC 3′		R‐This study	
Crust 16S	Crust16S_F(short)	Crustaceans	16S rRNA	5′ GGGACGATAAGACCCTATA 3′	~170	This study	51
	Crust16S_R(short)			5′ ATTACGCTGTTATCCCTAAAG 3′			
Mam 16S	16Smam1 (F)	Mammals	16S rRNA	5′ CGGTTGGGGTGACCTCGGA 3′	~90	Taylor (1996)	57
	16Smam2 (R)			5′ GCTGTTATCCCTAGGGTAACT 3′			
S_Ceph 16S	S_Cephalopoda_F	Cephalopods	16S rRNA	5′ GCTRGAATGAATGGTTTGAC 3′	~70	Peters et al. (2014)	50
	S_Cephalopoda_R			5′ TCAWTAGGGTCTTCTCGTCC 3′			

“F” refers to the forward primer; “R” refers to the reverse primer.

### 2.3 DNA extraction and quantification

Scats were subsampled (100–290 mg) and the DNA was extracted using the QIAmp Stool Mini Kit (Qiagen, CA, USA), following the manufacturer's instructions but using an overnight digestion at 55°C, 0.5× InhibitEX tablet, and eluting in 50 μl of AE Buffer. Extracts were diluted (1/5 and 1/20) in order to assess assay response, and amplification efficiency and inhibition using quantitative PCR (qPCR). All qPCR reactions were carried out in 25 μl consisting of final concentrations of: 1× Taq Gold buffer (Applied Biosystems [ABI], USA), 2 mmol/L MgCl_2_ (ABI, USA), 0.4 mg/ml BSA (Fisher Biotec, Australia), 0.25 mmol/L dNTPs (Astral Scientific, Australia), 0.4 μmol/L each of forward and reverse primers (Integrated DNA Technologies, Australia), 0.6 μl of 1/10,000 SYBR Green dye (Life Technologies, USA), 1 U of Taq polymerase Gold (ABI, USA), 2 μl of DNA, and made to volume with ultrapure water.

Each qPCR was run on a Step‐ONE qPCR thermocycler (ABI, USA) under the following conditions: 95°C for 5 min, followed by 50 cycles of 95°C for 30 s, 54–58°C for 30 s (the annealing temperature of each primer set is represented in Table 1) and 72°C for 45 s and a final extension of 10 min at 72°C. Where qPCR of an extract produced results in response to an assay, the DNA dilution with the highest relative proportion of starting template that showed uninhibited amplification (determined by qPCR *C*_T_ values) was selected for subsequent metabarcoding using assay‐specific fusion tagged primers (The number of PCR‐positive samples from each site and assay are shown in Table A3). The optimization of input DNA in amplicon sequencing workflows has been shown previously to benefit the sensitivity, reproducibility, and quality of metabarcoding data (Murray, Coghlan, & Bunce, 2015).

### 2.4 Library build and sequencing

Fusion tagged primers are gene‐specific primers which also incorporate MID (Multiplex IDentifier) tags of six to eight base pairs in length, and the appropriate Illumina/454 adaptor sequences. Unique combinations of these MID tags were assigned to each individual DNA extract to allow for the assignment of sequences to a sample postsequencing of pooled samples. To minimize cross‐contamination (in highly sensitive NGS workflows), no primer‐MID combination had been previously used, nor were combinations reused. Fusion PCR reactions were performed on DNA extracts (appropriate dilution determined by qPCR) in duplicate, and thermocycling conditions were used as described above. Tagged amplicons were purified using the Agencourt™ AMPure™ (Beckman Coulter Genomics, MA, USA) XP Bead PCR Purification kit as per the manufacturer's instructions, with the addition of a five‐minute incubation prior to elution at room temperature. The size and concentration of amplicons were estimated by electrophoresis on a 2% agarose gel stained with GelRed (Fisher Biotec, Australia), followed by visualization under UV light using a Bio‐Rad transilluminator**.**

Amplicons were combined in approximately equimolar concentrations to produce a single DNA library of all extracts for sequencing. The resultant library was purified as described above and quantified alongside a set of standard synthetic oligonucleotides of known molarity (Bunce, Oskam, & Allentoft, 2012) via qPCR, prior to sequencing (95°C for 5 min followed by 40 cycles of 95°C for 30 s and 60°C for 45 s). For the Mam 16S and Bird 12S assays, all sequencing was performed on Roche's 454 GS Junior (Lib A chemistry). For the remainder of the assays, sequencing was achieved using Illumina's MiSeq^®^ (300 cycle, version 2 reagent kit and Nano flow cell), following manufacturers protocols.

### 2.5 Data filtering and bioinformatics

Sequences were assigned to samples based on their MID tag using Geneious v.R8 (Kearse et al., 2012). As a method for quality control, only amplicons that contained a 100% nucleotide match to the MID, gene‐specific primer, and sequencing adapter regions were kept for further analysis (the number of reads passing this filter for each assay and per site is shown in Table A4). Adaptor/primer regions were removed, and the remaining amplicons were filtered using USEARCH's fastq filter with a maximum error of 0.5 (Edgar, 2010). The sequences were then separated into groups of unique sequences (these data are available for download on Data Dryad, https://doi.org/10.5061/dryad.rd748). Groups with sequence numbers of <1% of the total number of unique sequences detected in the sample were discarded in order to remove low‐abundant and potentially erroneous sequences (i.e., sequencing error and chimeras). Amplicons passing quality filtering were searched against the National Center for Biotechnology Information's (NCBI) GenBank nucleotide database (April 29 2015; Benson et al., 2015) using BLASTn (Basic Local Alignment Search Tool; Altschul, Gish, Miller, Myers, & Lipman, 1990) with the default parameters and a reward of 1. BLAST output files were imported into MEGAN (METaGenome ANalyzer; Huson, Mitra, Ruscheweyh, Weber, & Schuster, 2011) and visualized against the NCBI taxonomic framework using the LCA parameters: reporting of all reads, min bitscore 65.0, and reports limited to top 5% matches. Assignment of sequences to taxa was only considered where a match was made across the entire length of the query. Where further information was required regarding the habitat and commercialization of a species, the Atlas of Living Australia (2016) and FishBase (Froese & Pauly, 2016) were consulted (the number of reads assigned for each site and assay is shown in Table A4).

### 2.6 Operational taxonomic unit analysis

The operational taxonomic unit (OTU) analysis was performed using USEARCH (Edgar, 2010). Sequences were grouped into clusters (OTUs) using a 97% similarity threshold. The process also removed any chimeras, as well as clusters with a sequence abundance below 0.75% of the total number of unique sequences detected within the sample. Empirically these thresholds retained the sensitivity of the metabarcoding assays but removed low abundance OTUs that may be sequencing/PCR artifacts.

### 2.7 Statistical analysis

Despite the modest number of samples and sites, and the issues involving sampling times of the year, a statistical analysis was explored. Accordingly, a Jaccard dissimilarity index of the presence/absence data was performed in R (R Core Team, 2015) using the Vegan (Oksanen et al., 2016) and labdsv (Indval; Roberts, 2016) packages. A nested nonparametric (permutational) multivariate analysis of variance (adonis) was used to determine whether sea lion diet differed significantly between the five sampling areas nested within the Southern and Indian Oceans. A pairwise adonis with Holm correction (McLaughlin & Sainani, 2014) was also undertaken to ascertain the contribution of each site to the differences seen. The relationship of sampling sites was visualized using a nonmetric multidimensional scaling (nMDS). Finally, an estimate of indicator value (indval; Dufrêne & Legendre (1997)) was calculated to determine which taxa significantly influenced any differences observed in sea lion diet between oceans and among sites within each ocean. While it was tempting to investigate the relative abundance of NGS reads (within a PCR assay), the value of extracting quantitative data is questionable and unreliable. This is due to the variability in digestion rate and prey biomass, primer bias, mitochondrial molarity, and lack of conversion factors (Deagle et al., 2005; Thomas, Jarman, Haman, Trites, & Deagle, 2014). Accordingly, analyses were restricted to the presence/absence data.

## 3 RESULTS AND DISCUSSION

### 3.1 Overview of the results

**Figure 3:**
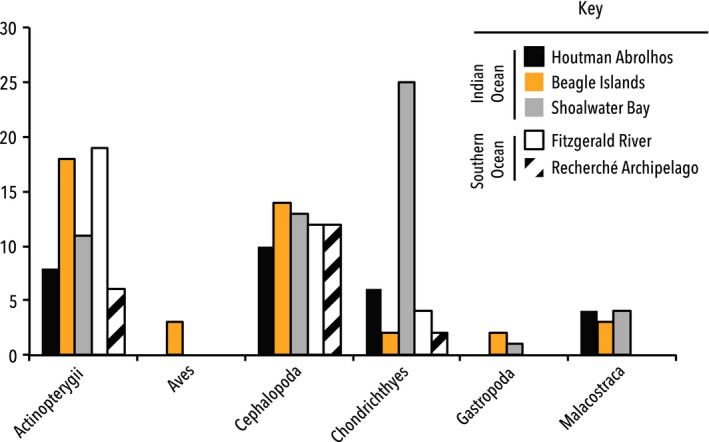
Sea lion diet: Classes of Taxa detected across the five WA study sites. The frequency a class of prey taxa was identified at each site using metabarcoding

The Mam 16S assay was used first to test whether the scat collected originated from an Australian sea lion. The remainder of the metabarcoding assays were used to determine the prey diversity found within the sea lion scats from each site. The taxa found belonged to six classes (Figure 3) from three phyla, representing over 20 orders and almost 40 families of prey.

The Mam 16S assay confirmed that 34 of the 36 beach‐collected samples originated from Australian sea lions (100% matches to reference *Neophoca cinerea* DNA sequences), many of these were later confirmed by the Plank COI assay. Of the two negative samples, one contained large amounts of human DNA while the other contained DNA that was amplified by the bird‐specific primers, potentially identifying the originator of the scat as *Pellicanus conspicillatus* (Australian pelican). These two samples were excluded from further analysis.

The nonmammalian metabarcoding assays were designed to characterize fish, crustacean, and cephalopod prey in these environmental samples. It is suggested that these assays will be useful for future metabarcoding studies on marine substrates such as scat, water, sediment, plankton tows, and gut contents.

Overall the multigene metabarcoding generated in excess of 1.2 million NGS reads, which were converted to the presence/absence data. These assays revealed (Figure 3) that while the majority of the sea lion samples (~68%) contained both ray‐finned fishes (Actinopterygii) and cephalopods (Cephalopoda), many sharks and rays (Chondrichthyes; ~22%) were also detected. This is especially true for those samples from Shoalwater Bay where Chondrichthyes made up the largest proportion of prey (~46%). The least common taxa were Aves and Gastropoda with only three detections each across the five sites. Table A3 shows the number of samples from each site that responded to each assay.

These findings are broadly consistent with the literature, although Kirkwood and Goldsworthy (2013) identify cephalopods as the top four sea lion prey items, followed by sharks and rays, lobsters and finally four species of ray‐finned fishes. However, their study concentrates on sea lions from South Australian waters where species composition will differ to those in the WA sites studied here. The Indian Ocean sites also contained 11 incidences of malacostracans (a class of crustaceans that includes crayfish and shrimp) and three of gastropods (a class of molluscs which contains bivalves), whereas these taxa were absent from the Southern Ocean sites.

The majority of the identified prey are benthic and are usually found at depths ≤150 m and most are found at <80 m. This finding concurs with studies that suggest the maximum foraging diving depth for an adult male sea lion is 150 m (Kirkwood & Goldsworthy, 2013).

### 3.2 Sea lion diet—Fish detections

Fish sequences were detected using both the Fish 16S and the Plank COI assays. Together, the two metabarcoding assays identified 47 Actinopterygii—36 of which were assigned to a genus or species level—and 17 Chondrichthyes—13 of which were ascribed to a genus or species level (Tables A5 and A6).

While there was some redundancy in the two assays that target fish, typically they detected different taxa; only five of the taxa were detected by both assays (Table A5). The Fish 16S assay detected 72% of the ray‐finned fishes compared with the Plank COI assay, which detected 38% of the ray‐finned fishes identified. For the cartilaginous fish, this trend was reversed, with the Fish 16S assay detecting 41% of the taxa identified and the Plank COI assay yielding 71%; only one genus (*Mustelus*) was detected by both assays (Table A6). These results demonstrate that, even with broad‐spectrum (“universal”) PCR assays, important species are still missed, and that when metabarcoding assays are used in combination, they yield far more information about the biodiversity of environmental samples. This is because the biotic “background” will vary between sites/samples and “generic” primers will exhibit sample dependent bias, where, due to primer binding variation, one group of taxa will preferentially amplify over another where they are both present in the sample (Pompanon et al., 2012). These biases are manifest further when samples are in low copy number and/or inhibited (Murray et al., 2015).

**Figure 4:**
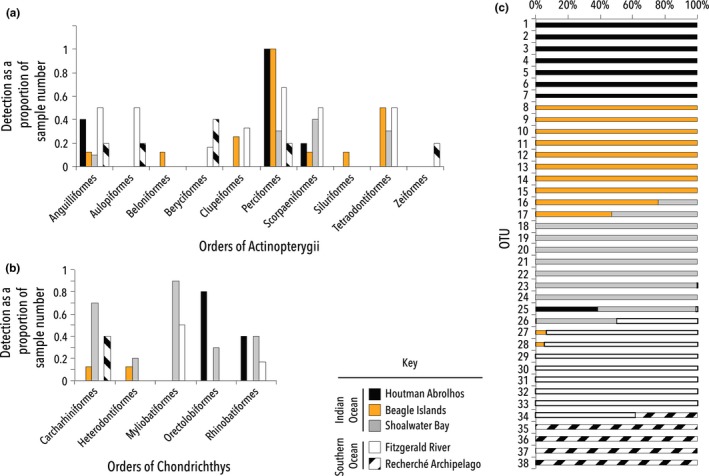
Metabarcoding of sea lion diet analyzed using ordinal and operational taxonomic unit (OTU) assignments. The number of times an order within (a) Actinopterygii and (b) Chondrichthyes was detected at each site as a proportion of the number of scat samples taken from each sample location. The OTU analysis of the Fish16S assay (c) demonstrates clear divisions between the genetic diversity of fish in the sample sites and between those samples sourced in the Indian Ocean compared with those from the Southern Ocean. The data used for (c) can be found in Table A9

Comparing sites, Perciformes were detected in all five samples from Houtman Abrolhos and the Beagle Islands, but were only detected in four of the six samples from Fitzgerald River, and were detected even less frequently in samples from Shoalwater Bay and Recherche Archipelago (Figure 4a). The order Perciformes contains a large variety of perch‐like fish including wrasse, parrotfish, goatfish, and damselfish. Fifteen taxa from this order were detected overall, with the vast majority of these identified from the Beagle Islands samples. The likely reason for this is that while Perciformes are found in all areas of southern Western Australia, the majority of those species detected in the sea lion scat are mainly found in the Indian Ocean. An example of this is *Pomacanthus semicirculatus*, which has only been recorded in northern waters of Australia (ALA, 2016). There is also a climatic shift from the Indian (warmer) and Southern Oceans (cooler) that may result in differences in prey species for sea lions. Tetraodontiformes, which includes the family Monacanthidae (leatherjackets), also seems to be favored across three sites (Beagle Island, Shoalwater Bay, and Fitzgerald River; Figure 4a). All these findings are in line with those of Peters et al. (2014), who also identified wrasse, goatfish, and leatherjackets as important prey for sea lions.

Of note is the detection of eels (Anguilliformes) as prey, by both the Fish and Plank COI assays. The species detected include the highfin moray (*Gymnothorax pseudothyrsoideus*), conger eels (*Conger* and *Gnathophis*), and unknown species of knot eels (in the Muraenidae family). The consumption of eels by the sea lions has not previously been reported, and yet the frequency of occurrence (eight samples across all five sites) suggests this is a regular component of sea lion diet.

In contrast to the other sites, a large proportion of sharks and rays are consumed by sea lions at Shoalwater Bay (Figure 4b). Each of the ten samples from Shoalwater contained prey from all five orders of Chondrochthyes detected, including stingarees (Urolophidae) and wobbegongs (Orectolobidae). Even in the Houtman Abrolhos Islands, four of the five samples produced sequences matching wobbegongs (Orectolobidae). While the Australian sea lion is known to eat sharks and rays (Kirkwood & Goldsworthy, 2013; Ling, 1992), it is suggested that many of the taxa identified here are previously unrecognized as sea lion prey.

The vast majority of the fishes detected in this study are classified as demersal or benthic and are found associated with reefs, seagrass, and the rocky and sandy bottoms of the continental shelf. This finding is consistent with current knowledge that describes the Australian sea lion as diving for its prey and being a principally benthic feeder (Gales & Cheal, 1992; Hesp et al., 2012; Kirkwood & Goldsworthy, 2013).

### 3.3 Sea Lion diet—Fish OTUs

There is a growing trend to move to taxonomic‐independent methods such as OTUs when describing genetic diversity in marine environments using metabarcoding data. This type of analysis allows for examination of all the available genetic diversity in metabarcoding data without the constraints of a frequently imprecise (and constantly evolving) taxonomic framework, coupled with an often‐incomplete collection of reference DNA barcodes.

Given the large number of fish taxa detected (Tables A5 and A6) using the Fish 16S metabarcoding assay, we analyzed the estimated genetic diversity of fish between sites using OTUs. After filtering, a total of 38 OTUs (Table A9; at 97% clustering) were identified from the 34 samples across the five study sites. Clear differences in regionality of fish diversity among sites were observed, with only seven of the 38 (~18%) OTUs shared across two or more sites (Figure 4c). When these sites were grouped by ocean (i.e., Southern or Indian Ocean), the division in genetic diversity was even more obvious, with only three of 38 (~8%) OTUs shared across the two oceans (Figure 4c).

From autumn to early spring (April to October), the Leeuwin Current (LC) brings warmer waters than would usually be found at these latitudes to the west coast of southern Western Australia (as well as tropical fish and invertebrates; Pearce & Feng, 2013), with the result that water temperatures are maintained at a warmer level during winter. While this current continues around to the southern coastline, it is supplemented by currents from subantarctic waters (Cresswell & Domingues, 2009), resulting in cooler environments in the Southern Ocean. Thus, the clear genetic distinction in the Fish OTUs between the oceans is likely attributable to these differences in the habitats; although we cannot rule out that temporal differences have also contributed.

### 3.4 Sea lion diet—Cephalopods and gastropods

Invertebrates, especially octopus, squid, and cuttlefish, are thought to make up a large proportion of the diet of the sea lions (Hesp et al., 2012; Kirkwood & Goldsworthy, 2013; McIntosh et al., 2006; Peters et al., 2014), but the actual invertebrate prey species remain largely unknown. The Plank COI, S_Ceph 16S, and Ceph 16S metabarcoding assays identified 14 invertebrate taxa, with 11 identified to a genus or species level (Table A7). However, many of the octopus species nominally identified have not previously been described in the collection area (those not known in Australia were assigned to higher taxa). This may be because the S_Ceph primer set target is a small amplicon (~70 bp), and therefore, one erroneous base, coupled with possible low interspecific variation at this locus, could result in erroneous assignments. The other possible reason is the relatively poor representation of the class on Genbank (of the taxa searched for in this study less than 75% had a 16S mtDNA sequence deposited in the database). However, as reference databases improve at widely utilized metabarcoding targets, so will our ability to make more robust taxonomic identifications.

**Figure 5:**
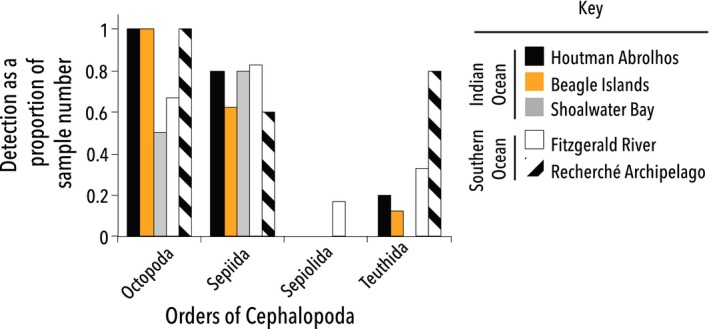
Sea lion diet: Orders of Cephalopod detected. The number of times an order within Cephalopoda was detected at each site as a proportion of the scat samples taken from each area. Data were obtained using the Ceph 16S, S_Ceph 16S, and Plank COI assays

Interestingly, the Plank COI assay also detected some cephalopods that provided additional support for taxa detected by both the Ceph and S_Ceph primers, in particular *Octopus* and *Sepia apama*. These two taxa were detected in 21 and 25 samples, respectively, and across all sites (Figure 5).

Of particular interest is the detection of the southern calamari squid (*S. australis*, order Teuthida), an important commercial species in Australia. While this species was detected in samples across four of the five sites (Figure 5), it was detected in less than a quarter of all samples (~18%), and in these samples, this was not the only prey revealed. This may indicate that the sea lions prefer octopus and giant cuttlefish to calamari, or it may suggest that the southern calamari squid is less abundant in the areas sampled. This latter possibility is perhaps more likely, as the occurrence records from the Atlas of Living Australia (2016) shows a decrease in the incidence of squid sightings west of the border with South Australia. Furthermore, in a South Australian sea lion study, Peters et al. (2014) also documented that *S. australis* is common prey.

The number of gastropod species detected was limited (Table A7) and these taxa have, to our knowledge, not been identified previously as potential sea lion prey. *Haliotis diversicolor* (many‐colored abalone) is found in the area where it was detected (ALA, 2016) and while *Stomatella impertusa* (False ear shell) was represented by only a few sequences in one sample, it does reside in Australian waters and the Genbank record had a 100% match with the queried sequence. Despite observing these taxa in more than one scat, it is difficult to exclude the possibility that the observations may be a consequence of secondary predation (the carryover of DNA from the gut of ingested prey species).

### 3.5 Sea lion diet—Crustaceans and birds

Crustaceans, including rock lobsters and swimming crabs, are noted as common prey of the Australian sea lion in South Australia (Kirkwood & Goldsworthy, 2013; McIntosh et al., 2006). The newly developed Crust16S assay detected five taxa, three to species level (Table A8). The results confirm that the Australian sea lion does prey on the commercially important western rock lobster (*Panulirus cygnus*), which was detected in six samples across all three sites in the Indian Ocean. This assay also detected a species of swimming crab (*Thalamita danae*) in a sample from the Houtman Abrolhos Islands; the only site where it is likely to be found (ALA, 2016).

The site at Shoalwater Bay is close to Penguin Island, which is home to a colony of little penguins (*Eudyptula minor*), a bird that is reported to be preyed upon by sea lions (McIntosh et al., 2006); as such, all samples were screened using the Bird 12S assay (which has been confirmed to detect *E. minor* in silico and in vivo), but no penguins were detected. However, we did detect the presence of one bird, a pied cormorant (*Phalocrocorax varius*), in three samples from Beagle Islands, which was also confirmed using the Plank COI assay. One of these samples also contained DNA from a bridled tern (*Onychoprion anaethetus*). While environmental contamination (e.g., sand on the beach, which was excluded as far as practicable) cannot be ruled out to explain the presence of both of these birds, neither can predation. Neither species of these birds has previously been documented as potential prey for Australian sea lions, but both are known to sit on the surface of the water (the pied cormorant also dives below the surface) and are thus susceptible to ambush predation from below.

Neither birds nor crustaceans were detected in the scats taken from the Southern Ocean sites. This may be because many of the crustaceans detected in the Indian Ocean are not known in the Southern Ocean, and while there are decapods in the Southern Ocean, they are not as prevalent as in other areas of Australia (ALA, 2016). However, as neither birds nor crustaceans appeared to make up a large proportion of the diet of the Indian Ocean sea lions, their absence in the diet of the Southern Ocean sea lions may be attributed merely to limited sample numbers, or prey preference at the time of sampling.

### 3.6 Spatial differences in sea lion diet

**Figure 6:**
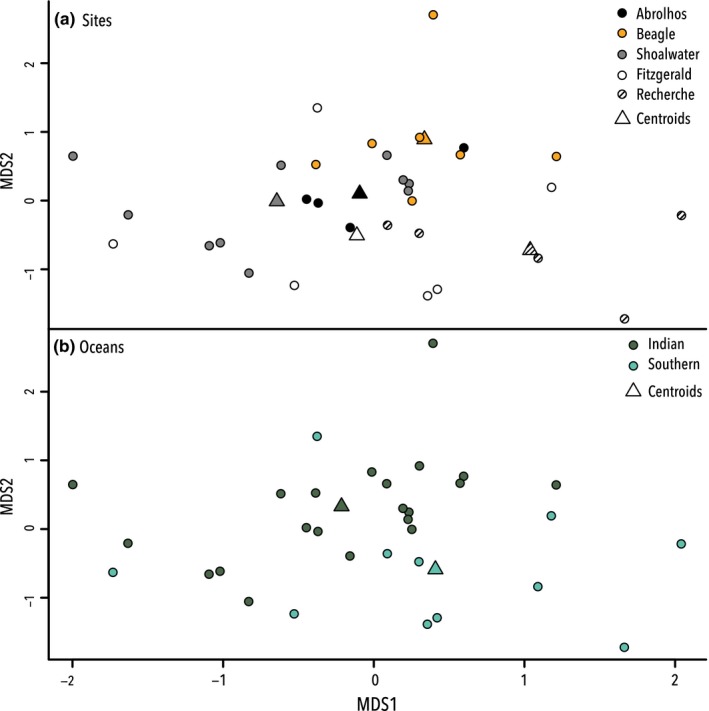
Multivariate analysis of all metabarcoding data assigned a taxonomic rank. (a) metaMDS plot comparing A taxa from the different sites of collection, and (b) the dietary differences between the sea lions of the Southern and Indian Oceans, centroids are marked with a triangles

The nested PERMANOVA (adonis) analysis showed that taxa preyed upon by sea lions were significantly different among Sites (*p* < .01) and between the Indian and Southern Ocean (*p* < .0001). A metaMDS plot (stress = 0.1595043) was used to visualize the differences in taxonomic assemblages among the five sampling sites and between the Indian and Southern Oceans (Figure 6). There was obvious clustering for the oceanic data; however, the distinctions between the individual sites were not as clear.

To investigate this, and despite the modest sample size, further PERMANOVA (adonis) analyses were conducted to explore potential differences within each ocean. These identified an overall significant difference between the three Indian Ocean sites (*p* < .007) but no significant variance among the two Southern Ocean sites. Subsequently, a pairwise adonis was used investigate which Indian Ocean sites were different; this revealed that the only significant difference was between Houtman Abrolhos and Beagle Islands (*p* < .05).

**Figure 7:**
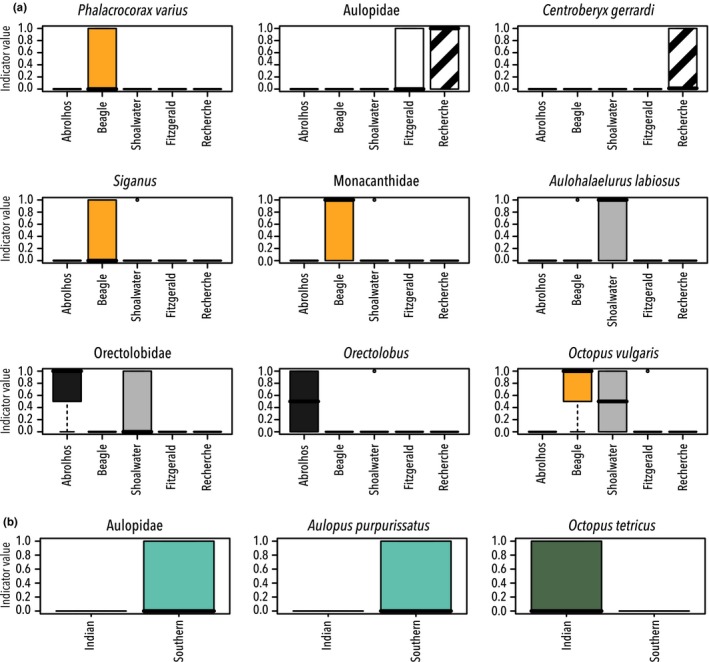
Indicator species analysis. Indval results from the total metabarcoding dataset showing the taxa characterizing each area and thus driving variations in sea lion diet between (a) sites, (b) oceans (all *p* values <.05)

To determine which taxa contributed to the significant differences in the PERMANOVA, indicator values analysis (indval) was performed. An indval analysis enables the taxa responsible for the regionality in the data to be discerned. While the 34 scats analyzed here are somewhat underpowered, the analysis is valuable due to the identification of taxa that drive the spatial patterns in the data. The indval analysis executed on the total metabarcoding dataset identified nine primary taxa that drive the variation in sea lion diet among sites (*p* = .005–.04), and three primary taxa that drive the differences in taxonomic assemblage observed between the Indian and Southern Ocean (*p* = .002–.04; Figure 7).

Given that birds and crustacea were only detected in the Indian Ocean, it may have been expected that these taxa would drive differences between the two oceans. However, this is not the case; in the Indian Ocean, it is *Octopus tetricus* that is flagged as a key indicator species and in the Southern Ocean it is fish, Aulopidae (threadsails) and in particular *Aulopus purpurissatus* (sergeant baker).

In the site indval analysis, Beagle Islands had four of the nine key indicator species (a bird (*P. varius*), some Actinopterygii (Monacanthidae and *Siganus*), and a species of *Octopus*. This is in keeping with the adonis analyses above, which showed Beagle Islands were significantly different from each of the other sites. Indicator taxa characterizing Shoalwater are predominantly carpet sharks, Orectolobidae, and *Aulohalaelurus labiosus*. Carpet sharks (Orectolobidae and *Orectolobus*) are also the key indicator species for the Houtman Abrolhos Islands.

Actinopterygii are the key indicator species in the Southern Ocean sites. Aulopidae are notable taxa at both Recherche and Fitzgerald, which is unsurprising as it was flagged as key indicator for differences found between the two oceans (Figure 7). In Recherche Archipelago, *Centroberyx gerrardi* (red snapper), a commercial species, was also identified as an indicator species; although it was only found in two of the five samples taken from the area.

## 4 CONCLUSION

This was the first attempt to investigate and describe the diet of the Australian sea lion by DNA metabarcoding of scats. Despite the relatively small number of scats analyzed here (*n* = 34), the results demonstrate the sensitivity of the approach to identify previously unrecorded species such as eels, gastropods, and the frequency of sharks and rays in the diet. Importantly, metabarcoding offers a different method allowing identification taxa that are either difficult to detect through morphological analysis of feces or through direct observational studies. This study, like previous dietary studies using metabarcoding, have been somewhat hampered by lack of reference barcodes, but despite this limitation, the dietary audit presented here presents significant insight into the prey of this apex predator. Significantly, the comprehensiveness of these datasets will improve with time, and environmental data, such as generated here, can be re‐analyzed. Finally, the data gathered from the scat of this endangered apex predator demonstrate that DNA metabarcoding is a relatively simple and noninvasive way to both monitor the sea lions’ diet and to provide valuable insights into the regional biodiversity of our oceans. It is foreseen that the expansion of this type of project both temporally and spatially can only add to the information gathered presented here.

Less than half of the marine species detected in this dietary study are classified as commercial species (ALA, 2016; Fishbase (Froese & Pauly, 2016)). While it is clear the sea lions are preying on some commercial species (such as the commercially important western rock lobster, *P. cygnus*, and southern calamari squid, *S. australis*), sea lions are taking a large variety of prey and no particular commercial species seems to dominate their diet. The diversity of taxa exploited by the Australian sea lion between oceans, sites, and even between samples supports the notion that Australian sea lions are opportunistic feeders. This bodes well for the survival of this protected species, as (providing its core habitats are preserved), its mode of feeding makes it more likely to adapt its diet to changes in the surrounding biodiversity.

## CONFLICT OF INTEREST

None declared.

## APPENDIX 1

### 1.1

**Table A1:** Sample collection data; details of collection dates and sites and number of scats collected

Reserve	Ocean	No. samples	Date collected
Houtman Abrolhos Nature Reserve	Indian	5	10 April 2013
Beagle Islands Nature Reserve	Indian	8	17 May 2013
Shoalwater Islands Nature Reserve	Indian	1	October 2012
		5	20/21 January 2013
		4	22 May 2013
Fitzgerald River Nature Reserve	Southern	6	October 2012
Recherche Archipelago Nature Reserve	Southern	4	26 January 2013
		1	27 January 2013

**Table A2:** Single source analysis of metabarcoding assays; details of assays tested against DNA extracted from single source samples (barcode size in brackets)

Class	Assignment	Metabarcoding assay and % match of query to reference			
		Crust (170 bp)	Fish (200 bp)	S_Ceph (70 bp)	Ceph (200 bp)
Actinopterygii	*Encrasicholina punctifer*		99–100		
	*Hyporhamphus melanochir*		99–100		
	*Sardinops* (*sagax*/*neopilchardus*)		99–100		
	*Spratelloide srobustus*		99–100		
Malacostraca	*Fenneropenaeus merguiensis*	99–100			
	*Portunus pelagicus*	99–100			
Cephalopoda	*Nototodarus sloanii*				98–100
	Ommastrephidae (*Martialia hyadesi*/*Nototodarus sloanii*/*Todarodes filippovae*)			97–100	

**Table A3:** Number of samples producing results for each assay; the total number of samples from each site is in brackets

Assay	Houtman Abrolhos (6)	Beagle (8)	Shoalwater Bay (10)	Fitzgerald (6)	Recherche Archipelago (5)
	Number of samples producing results				
Bird 12S	0	3	0	0	0
Ceph 16S	4	6	9	4	1
Crust 16S	3	3	5	0	0
Fish 16S	4	5	8	4	3
Mam 16S	6	8	10	6	5
Plank COI	5	8	10	5	4
S_Ceph 16S	4	6	9	6	5

**Table A4:** Numbers of sequences per assay, per site; “Unfiltered” refers to sequences that have been 100% matched to the sequence specific primers, the MID tags, and the adaptor sequence

Site	Sequence type	Ceph 16S	S_Ceph 16S	Fish 16S	Plank COI	Crust 16S
Houtman Abrolhos	Unfiltered	36225	114540	56420	30530	34167
	Mean unique	395 ± 431	694 ± 453	1487 ± 367	324 ± 138	622 ± 504
	Filtered and assigned	33164	108929	42584	28499	30999
	Mean unique	55 ± 16	95 ± 13	34 ± 25	65 ± 25	34 ± 12
Beagle Islands	Unfiltered	23183	147703	73954	42126	30913
	Mean unique	327 ± 403	555 ± 208	1685 ± 479	309 ± 126	898 ± 518
	Filtered and assigned	19909	140073	53570	38603	25559
	Mean unique	35 ± 14	88 ± 23	25 ± 4	77 ± 32	47 ± 21
Shoalwater Bay	Unfiltered	34613	146541	92175	52095	81945
	Mean unique	331 ± 250	479 ± 331	1385 ± 745	259 ± 148	862 ± 266
	Filtered and assigned	29110	125647	50835	41130	41899
	Mean unique	46 ± 19	81 ± 22	26 ± 18	43 ± 18	51 ± 8
Fitzgerald River	Unfiltered	7754	50363	94898	15122	0
	Mean unique	168 ± 125	368 ± 143	2301 ± 742	198 ± 113	0
	Filtered and assigned	3624	45758	57549	14045	0
	Mean unique	59 ± 47	74 ± 11	37 ± 25	60 ± 40	0
Recherche Archipelago	Unfiltered	332	55926	36321	20501	0
	Mean unique	50	534 ± 124	1239 ± 165	218 ± 160	0
	Filtered and assigned	325	46432	27500	19472	0
	Mean unique	48	62 ± 31	29 ± 13	62 ± 42	0

“Filtered and assigned” refers to the number of sequences that have passed through the Usearch filtering process and were assigned to taxa. “Mean unique” refers to the mean number of unique sequences produced given the number of positive samples for the assay.

**Table A5:** Actinopterygii (ray‐finned fishes) identified with Plank COI and Fish 16S assays

Order	Family	Genus/species	Common name	Abrolhos	Beagle	Shoalwater	Fitzgerald	Recherche
Anguilliformes	Congridae	*Conger*	Genus of Conger eels		Plank (1)	Plank (1)	Plank (2), Fish (2)	
		*Gnathophis*	Genus of Conger eels				Fish (1)	
	Muraenidae		Knot‐eels	Fish (1)				Fish (1)
		*Gymnothorax pseudothyrsoideus*	Highfin Moray	Fish (1)				
Aulopiformes	Aulopidae		Aulopus				Plank (2)	Plank (3)
		*Aulopus purpurissatus*	Sergeant Baker				Fish (3)	Fish (1)
Beloniformes	Exocoetidae		Flying fishes		Fish (1)			
Beryciformes	Anoplogastridae		Fangtooths				Fish (1)	
	Berycidae	*Centroberyx australis*	Yellow‐eyed Red Snapper					Fish (1)
		*Centroberyx gerrardi*	Red Snapper					Plank (2)
Clupeiformes	Clupeidae		Herrings		Fish (1)			
		*Etrumeus*	Maray				Plank (1)	
		*Sardinops sagax*	Australian Sardine				Fish (1)	
	Engraulidae	*Engraulis*	Anchovies		Fish (1)			
Perciformes			Perch‐like Fishes			Fish (1)		
	Labridae		Wrasses		Fish (1)			
		*Coris auricularis*	Western King Wrasse		Plank (1)			
		*Leptoscarus vaigiensis*	Marbled Parrotfish	Fish (1)				
		*Notolabrus*	Wrasses		Plank (2)			
		*Odax acroptilus*	Marine Rainbowfish		Fish (1)			
		*Odax cyanomelas*	Herring Cale				Plank(2), Fish (1)	
	Mullidae	*Parupeneus*	Common Goatfish	Fish (1)				
		*Parupeneus spilurus*	Blacksaddle Goatfish	Plank (1)				
		*Upeneichthys*	Goatfish		Fish (1)	Fish (1)	Fish (1)	
		*Upeneichthys stotti*	Stott's Goatfish		Fish (1)		Fish (1)	
	Pempheridae	*Parapriacanthus*	Bullseyes				Fish (1)	
	Pomacanthidae	*Pomacanthus semicirculatus*	Blue Angelfish	Fish (1)				
	Pomacentridae		Damselfishes	Plank (2)	Plank (1)			
		*Chromis*	Damselfishes		Fish (1)			
		*Parma microlepis*	White Ear		Fish (1)			
	Siganidae	*Siganus*	Rabbitfish		Fish (2), Plank (2)	Fish (1)		
Scorpaeniformes			Scorpion Fishes & Sculpins				Fish (1)	
	Platycephalidae	*Leviprora inops*	Crocodile Flathead	Plank (2)	Plank (1)	Plank (4)		
		*Platycephalus aurimaculatus*	Tiger Flathead				Plank (2)	
		*Platycephalus longispinis*	Long‐spine Flathead				Plank (1)	
	Scorpaenidae	*Scorpaenodes*	Scorpion Fish				Fish (1)	
Siluriformes	Plotosidae		Blunt‐tail Catfishes		Plank (1)			
Tetraodontiformes	Monacanthidae		Leatherjackets		Fish (1)			
		*Chaetodermis penicilligera*	Tasselled Leatherjacket		Fish (1), Plank (1)	Fish (2), Plank (1)		
		*Eubalichthys mosaicus*	Mosaic Leatherjacket		Fish (1)			
		*Monacanthus chinensis*	Fanbelly Leatherjacket		Fish (1)			
		*Nelusetta ayraudi*	Ocean Jacket				Plank (1)	
		*Scobinichthys granulatus*	Rough Leatherjacket		Fish (1), Plank (2)			
		*Thamnaconus*	Leatherjacket			Fish (1)		
	Tetraodontidae	*Omegophora*	Toadfish				Fish (1)	
		*Omegophora armilla*	Ringed Toadfish				Plank (2)	
Zeiformes	Zeidae	*Zeus faber*	John Dory					Fish (1)

The number of samples in which the taxa were detected is indicated in the brackets.

**Table A6:** Chondritchthyes (sharks and rays) identified with Plank COI and Fish 16S assays

Order	Family	Genus/species	Common name	Abrolhos	Beagle	Shoalwater	Fitzgerald	Recherche
Carcharhiniformes			Ground sharks			Fish (2)		Plank (1)
	Scyliorhinidae	*Aulohalaelurus labiosus*	Black Spotted Catshark		Plank (1)	Plank (6)		
	Triakidae	*Mustelus*	Gummy shark					Fish (1), Plank (1)
Heterodontiformes	Heterodontidae	*Heterodontus*				Fish (1)		
		*Heterodontus portusjacksoni*	Port Jackson Shark		Plank (1)	Plank (2)		
Myliobatiformes	Myliobatidae	*Myliobatis australis*	Southern Eagle Ray			Plank (1)		
	Urolophidae		Stingarees/Round Rays			Fish (1)		
		*Trygonoptera*				Plank (1)	Plank (1)	
		*Trygonoptera mucosa*	Western Shovelnosed Stingaree				Plank (1)	
		*Trygonoptera personata*	Masked Stingaree			Plank (3)	Plank (1)	
		*Urolophus lobatus*	Lobed Stingaree			Plank (3)	Plank (1)	
		*Urolophus paucimaculatus*	Sparsely Spotted Stigaree			Plank (1)	Plank (1)	
Orectolobiformes	Orectolobidae		Wobbegongs	Plank (4)		Plank (3)		
		*Orectolobus*		Fish (2)		Fish (2)		
Rhinobatiformes	*Rhinobatidae*		Guitar fishes	Fish (1)		Fish (2)		
		*Aptychotrema vincentiana*	Western Shovelnose Ray	Plank (1)		Plank (1)		
		*Trygonorrhina guanerius*	Southern Fiddler Ray			Fish (1)		

The number of samples in which the taxa was detected is indicated in the brackets.

**Table A7:** Cephalopod and Gastropod taxa identified with Ceph 16S, S_Ceph 16S, and Plank COI assays

Class	Order	Family	Genus/species	Common name	Abrolhos	Beagle	Shoalwater	Fitzgerald	Recherche
Cephalopoda	Octopodiformes	Octopodidae		Octopus	S_Ceph (2)	S_Ceph (8)	S_Ceph (1)	S_Ceph (2) Ceph (1)	
			*Grimpella thaumastocheir**	Velvet Octopus	S_Ceph (1)	S_Ceph (2)			
			*Octopus*		S_Ceph (4), Plank (2), Ceph (1)	S_Ceph (6), Plank (3)	S_Ceph (5), Plank (4)	S_Ceph (2)	S_Ceph (2)
			*Octopus ornatus**		Ceph (1)	Ceph (1)			
			*Octopus tetricus*	Gloomy Octopus	S_Ceph (1)	S_Ceph (3)	S_Ceph (5)		
			*Octopus vulgaris**	Common Octopus		S_Ceph (3), Ceph (4)	S_Ceph (4), Ceph (5)	Ceph (1)	
			*Scaeurgus*					S_Ceph (1)	
	Oegopsida	Ommastrephidae	*Nototodarus gouldi*/*Todarodes pacificus**						S_Ceph (2)
			*Nototodarus gouldi*	Red Arrow Squid					Ceph (1)
	Myopsida	Loliginidae	*Sepioteuthis australis*	Southern Calamari Squid	S_Ceph (1), Ceph (1)	S_Ceph (1), Ceph (1)		S_Ceph (2), Ceph (1)	S_Ceph (2)
	Sepiida	Sepiidae		Cuttlefish					S_Ceph (1), Plank(1)
			*Sepia apama*	Giant Cuttlefish	S_Ceph(4), Plank (4), Ceph (4)	S_Ceph (5), Plank (3), Ceph (5)	S_Ceph (8), Plank (5), Ceph (7)	S_Ceph (5), Plank (4), Ceph (1)	S_Ceph (2)
		Sepiolidae		Dumpling Squids				S_Ceph (1)	
Gastropoda		Haliotidae	*Haliotis*			S_Ceph (1)	S_Ceph (1), Ceph (1)		
			*Haliotis diversicolor*	Many Colored Abalone		S_Ceph (2)			
		Trochidae	*Stomatella impertusa*	False ear shell				S_Ceph (1)	

The number of samples in which the taxa were detected is indicated in the brackets. Those species found in Australia, but not in the area of collection, are indicated by an asterisk (*).

**Table A8:** Crustaceans and Aves detected using Crust 16S, Bird 12S, and Plank COI assays

Class	Order	Family	Genus/species	Common name	Abrolhos	Beagle	Shoalwater	Fitzgerald	Recherche
Malacostraca	Decapoda			Crustaceans			Crust (1)		
		Palinuridae	*Panulirus cygnus*	Western Rock Lobster	Crust (1)	Crust (3)	Crust (2)		
		Portunoidea	*Thalamita danae*	Unknown Swimmer Crab	Crust (1)				
		Xanthidae		Crabes de Boue	Crust (1)				
Aves	Charadriformes	Laridae	*Onychoprion anaethetus*	Bridled Tern		Plank (1)			
	Suliformes	Phalacrocoracidae	*Phalacrocorax varius*	Pied Cormorant		Plank (2), Bird (3)			

The number of samples in which the taxa were detected is indicated in the brackets.

**Table A9:** Fish 16S OTU sequence abundance per site and per ocean

OTU ID	Abrolhos	Beagle	Shoalwater	Fitzgerald	Recherche	Indian Ocean	Southern Ocean
OTU_1	13,242	0	0	0	0	13,242	0
OTU_7	7,980	0	0	0	0	7,980	0
OTU_16	8,964	0	0	0	0	8,964	0
OTU_29	1,532	0	0	0	0	1,532	0
OTU_30	1,441	0	0	0	0	1,441	0
OTU_34	1,536	0	0	0	0	1,536	0
OTU_38	267	0	0	0	0	267	0
OTU_6	0	8,456	0	0	0	8,456	0
OTU_12	0	7,816	0	0	0	7,816	0
OTU_19	0	4,092	0	0	0	4,092	0
OTU_21	0	3,106	0	0	0	3,106	0
OTU_27	0	2,165	0	0	0	2,165	0
OTU_28	0	1,685	0	0	0	1,685	0
OTU_31	0	1,160	0	0	0	1,160	0
OTU_35	0	851	0	0	0	851	0
OTU_8	0	11,384	3,620	0	0	15,004	0
OTU_10	0	8,972	10,141	0	0	19,113	0
OTU_14	0	0	5,014	0	0	5,014	0
OTU_15	0	0	7,118	0	0	7,118	0
OTU_17	0	0	4,395	0	0	4,395	0
OTU_20	0	0	3,137	0	0	3,137	0
OTU_22	0	0	2,719	0	0	2,719	0
OTU_23	0	0	5,008	4	0	5,008	4
OTU_25	0	0	2,386	0	0	2,386	0
OTU_11	4,329	0	6,858	96	0	11,187	96
OTU_5	0	46	10,736	10,787	0	10,782	10,787
OTU_36	0	117	0	1,659	0	117	1,659
OTU_37	0	17	0	315	0	17	315
OTU_3	0	0	0	11,830	0	0	11,830
OTU_4	0	0	0	11,695	0	0	11,695
OTU_13	0	0	0	7,989	0	0	7,989
OTU_24	0	0	0	2,414	0	0	2,414
OTU_32	0	0	0	1,913	0	0	1,913
OTU_2	0	0	0	16,951	10,632	0	27,583
OTU_9	0	0	0	0	7,835	0	7,835
OTU_18	0	0	0	0	4,375	0	4,375
OTU_26	0	0	0	0	2,016	0	2,016
OTU_33	0	0	0	0	1,011	0	1,011

## References

[ece33123-bib-0001] ALA (2016). Atlas of Living Australia. Retrieved from http://www.ala.org.au

[ece33123-bib-0002] Altschul, S. F. , Gish, W. , Miller, W. , Myers, E. W. , & Lipman, D. J. (1990). Basic local alignment search tool. Journal of Molecular Biology, 215, 403–410. 2231712 10.1016/S0022-2836(05)80360-2

[ece33123-bib-0003] Benson, D. A. , Clark, K. , Karsch‐Mizrachi, I. , Lipman, D. J. , Ostell, J. , & Sayers, E. W. (2015). GenBank. Nucleic Acids Research, 43(Database issue), D30–D35. http://doi.org/10.1093/nar/gku1216. 25414350 10.1093/nar/gku1216 PMC4383990

[ece33123-bib-0004] Berry, O. , Bulman, C. , Bunce, M. , Coghlan, M. , Murray, D. C. , & Ward, R. D. (2015). Comparison of morphological and DNA metabarcoding analyses of diets in exploited marine fishes. Marine Ecology Progress Series, 540, 167–181.

[ece33123-bib-0005] Boyer, S. , Cruickshank, R. H. , & Wratten, S. D. (2015). Faeces of generalist predators as ‘biodiversity capsules’: A new tool for biodiversity assessment in remote and inaccessible habitats. Food Webs, 3, 1–6.

[ece33123-bib-0006] Brown, D. S. , Jarman, S. N. , & Symondson, W. O. C. (2012). Pyrosequencing of prey DNA in reptile faeces: Analysis of earthworm consumption by slow worms. Molecular Ecology Resources, 12, 259–266. 22176947 10.1111/j.1755-0998.2011.03098.x

[ece33123-bib-0007] Bunce, M. , Oskam, C. , & Allentoft, M. (2012). Quantitative real‐time PCR in aDNA research In Shapiro B. & Hofreiter M. (Eds.), Ancient DNA: Methods and protocols, methods in molecular biology (pp. 121–132). New York City, New York: Springer Science+Business Media. 10.1007/978-1-61779-516-9_16 22237530

[ece33123-bib-0008] Casper, R. M. , Jarman, S. N. , Gales, N. J. , & Hindell, M. A. (2007). Combining DNA and morphological analyses of faecal samples improves insight into trophic interactions: A case study using a generalist predator. Marine Biology, 152, 815–825.

[ece33123-bib-0009] Cooper, A. (1994). DNA from museum specimens. New York, NY: Springer.

[ece33123-bib-0010] Costa, D. P. , & Gales, N. J. (2003). Energetics of a benthic diver: Seasonal foraging ecology of the Australian sea lion, *Neophoca Cinerea* . Ecological Monographs, 73, 27–43.

[ece33123-bib-0011] Cresswell, G. , & Domingues, C. M. (2009). Leeuwin current A2 In Steele J. H. (Ed.), Encyclopedia of ocean sciences (2nd ed., pp. 444–454). Oxford, UK: Academic Press.

[ece33123-bib-0012] Deagle, B. E. , Gales, N. J. , Evans, K. , Jarman, S. N. , Robinson, S. , Trebilco, R. , & Hindell, M. A. (2007). Studying seabird diet through genetic analysis of faeces: A case study on macaroni penguins (*Eudyptes chrysolophus*). PLoS One, 2, e831. 17786203 10.1371/journal.pone.0000831 PMC1959119

[ece33123-bib-0013] Deagle, B. E. , Kirkwood, R. , & Jarman, S. N. (2009). Analysis of Australian fur seal diet by pyrosequencing prey DNA in faeces. Molecular Ecology, 18, 2022–2038. 19317847 10.1111/j.1365-294X.2009.04158.x

[ece33123-bib-0014] Deagle, B. E. , Tollit, D. J. , Jarman, S. N. , Hindell, M. A. , Trites, A. W. , & Gales, N. J. (2005). Molecular scatology as a tool to study diet: Analysis of prey DNA in scats from captive Steller sea lions. Molecular Ecology, 14, 1831–1842. 15836654 10.1111/j.1365-294X.2005.02531.x

[ece33123-bib-0015] Delport, T. C. , Power, M. L. , Harcourt, R. G. , Webster, K. N. , & Tetu, S. G. (2016). Colony location and captivity influence the gut microbial community composition of the Australian sea lion (*Neophoca cinerea*). Applied and Environmental Microbiology, 82, 3440–3449. 27037116 10.1128/AEM.00192-16 PMC4959163

[ece33123-bib-0500] Dufrêne, M ., & Legendre, P. (1997). Species assemblages and indicator species: the need for a flexible asymmetrical approach. Ecological Monographs, 67, 345–366.

[ece33123-bib-0016] Edgar, R. C. (2010). Search and clustering orders of magnitude faster than BLAST. Bioinformatics, 26, 2460–2461. 20709691 10.1093/bioinformatics/btq461

[ece33123-bib-0017] Froese, R. , & Pauly, D. (2016). Fishbase. World Wide Web electronic publication. Retrieved from http://www.fishbase.org

[ece33123-bib-0018] Gales, N. J. , & Cheal, A. J. (1992). Estimating diet composition of the Australian sea‐lion (*Neophoca‐Cinerea*) from scat analysis – An unreliable technique. Wildlife Research, 19, 447–456.

[ece33123-bib-0019] Goldsworthy, S. D. (2015). Neophoca cinerea, Australian Sea Lion, The IUCN Red List of Threatened Species. https://doi.org/10.2305/iucn.uk.2015-2.rlts.t14549a45228341.en

[ece33123-bib-0020] Goldsworthy, S. , McKenzie, J. , Shaughnessy, P. , McIntosh, R. , Page, B. , & Campbell, R. (2009). An update of the report: Understanding the impediments to the growth of Australian sea lion populations. SARDI research report series.

[ece33123-bib-0021] Hardy, N. , Berry, T. , Kelaher, B. P. , Goldsworthy, S. D. , Bunce, M. , Coleman, M. A. , … Figueira, W. (In press). Assessing the trophic ecology of top predators across a recolonisation frontier using DNA metabarcoding of diets. Marine Ecology Progress Series, https://doi.org/10.3354/meps12165

[ece33123-bib-0022] Hesp, S. A. , Tweedley, J. R. , McAuley, R. , Tink, C. J. , Campbell, R. A. , Chuwen, B. M. , & Hall, N. G. (2012). Informing tisk assessment through estimating interaction rates between Australian sea lions and Western Australia's temperate demersal gillnet fisheries, Fisheries Research and Development Corporation Report. Murdoch: Centre for Fish and Fisheries Research, Murdoch University.

[ece33123-bib-0023] Hibert, F. , Taberlet, P. , Chave, J. , Scotti‐Saintagne, C. , Sabatier, D. , & Richard‐Hansen, C. (2013). Unveiling the diet of elusive rainforest herbivores in next generation sequencing era? The tapir as a case study. PLoS One, 8(4), e60799. 23560107 10.1371/journal.pone.0060799 PMC3613382

[ece33123-bib-0024] Huson, D. H. , Mitra, S. , Ruscheweyh, H. J. , Weber, N. , & Schuster, S. C. (2011). Integrative analysis of environmental sequences using MEGAN 4. Genome Research, 21, 1552–1560. 21690186 10.1101/gr.120618.111 PMC3166839

[ece33123-bib-0025] Kearse, M. , Moir, R. , Wilson, A. , Stones‐Havas, S. , Cheung, M. , Sturrock, S. , … Drummond, A. (2012). Geneious Basic: An integrated and extendable desktop software platform for the organization and analysis of sequence data. Bioinformatics, 28, 1647–1649. 22543367 10.1093/bioinformatics/bts199 PMC3371832

[ece33123-bib-0026] Kirkwood, R. , & Goldsworthy, S. (2013). Fur Seals and Sea Lions. Collingwood, Vic.: CSIRO Publishing.

[ece33123-bib-0027] Ling, J. K. (1992). *Neophoca cinerea* . Mammalian Species, 392, 1–7.

[ece33123-bib-0028] McIntosh, R. R. , Page, B. , & Goldsworthy, S. D. (2006). Dietary analysis of regurgitates and stomach samples from free‐living Australian sea lions. Wildlife Research, 33, 661–669.

[ece33123-bib-0029] McLaughlin, M. J. , & Sainani, K. L. (2014). Bonferroni, Holm, and Hochberg corrections: Fun names, serious changes to p values. PM R, 6, 544–546. 24769263 10.1016/j.pmrj.2014.04.006

[ece33123-bib-0030] Murray, D. C. , Bunce, M. , Cannell, B. L. , Oliver, R. , Houston, J. , White, N. E. , … Haile, J. (2011). DNA‐based faecal dietary analysis: A comparison of qPCR and high throughput sequencing approaches. PLoS One, 6(10), e25776. 21998697 10.1371/journal.pone.0025776 PMC3188572

[ece33123-bib-0031] Murray, D. C. , Coghlan, M. L. , & Bunce, M. (2015). From benchtop to desktop: Important considerations when designing amplicon sequencing workflows. PLoS One, 10, e0124671. 25902146 10.1371/journal.pone.0124671 PMC4406758

[ece33123-bib-0032] Oksanen, J. , Guillaume Blanchet, F. , Friendly, M. , Kindt, R. , Legendre, P. , McGlinn, D. , … Wagner, H. (2016). vegan: Community Ecology Package. R package version 2.3‐0. Retrieved from http://CRAN.R-project.org/package=vegan, in: Oksanen, J. (Ed.).

[ece33123-bib-0033] Osterrieder, S. K. , Salgado Kent, C. , & Robinson, R. W. (2015). Variability in haul‐out behaviour by male Australian sea lions *Neophoca cinerea* in the Perth metropolitan area, Western Australia. Endangered Species Research, 28, 259–274.

[ece33123-bib-0034] Osterrieder, S. K. , Salgado Kent, C. , & Robinson, R. W. (2016). Responses of Australian sea lions, *Neophoca cinerea*, to anthropogenic activities in the Perth metropolitan area, Western Australia. Aquatic Conservation: Marine and Freshwater Ecosystems, 27, 414–435.

[ece33123-bib-0035] Pearce, A. F. , & Feng, M. (2013). The rise and fall of the “marine heat wave” off Western Australia during the summer of 2010/2011. Journal of Marine Systems, 111–112, 139–156.

[ece33123-bib-0036] Peters, K. J. , Ophelkeller, K. , Bott, N. J. , Deagle, B. E. , Jarman, S. N. , & Goldsworthy, S. D. (2014). Fine‐scale diet of the Australian sea lion (*Neophoca cinerea*) using DNA‐based analysis of faeces. Marine Ecology, 36, 347–367.

[ece33123-bib-0037] Peters, K. J. , Ophelkeller, K. , Bott, N. J. , & Goldsworthy, S. D. (2015). PCR‐based techniques to determine diet of the Australian sea lion (*Neophoca cinerea*): A comparison with morphological analysis. Marine Ecology, 36, 1428–1439.

[ece33123-bib-0038] Pompanon, F. , Deagle, B. E. , Symondson, W. O. C. , Brown, D. S. , Jarman, S. N. , & Taberlet, P. (2012). Who is eating what: Diet assessment using next generation sequencing. Molecular Ecology, 21, 1931–1950. 22171763 10.1111/j.1365-294X.2011.05403.x

[ece33123-bib-0039] Quemere, E. , Hibert, F. , Miquel, C. , Lhuillier, E. , Rasolondraibe, E. , Champeau, J. , … Chikhi, L. (2013). A DNA metabarcoding study of a primate dietary diversity and plasticity across its entire fragmented range. PLoS One, 8, e58971. 23527060 10.1371/journal.pone.0058971 PMC3602585

[ece33123-bib-0040] R Core Team (2015). R: A language and environment for statistical computing. Vienna, Austria: R Foundation for Statistical Computing.

[ece33123-bib-0041] Radulovici, A. E. , Sainte‐Marie, B. , & Dufresne, F. (2009). DNA barcoding of marine crustaceans from the Estuary and Gulf of St Lawrence: A regional‐scale approach. Molecular Ecology Resources, 9, 181–187. 21564977 10.1111/j.1755-0998.2009.02643.x

[ece33123-bib-0042] Roberts, D. W. (2016). labdsv: Ordination and Multivariate Analysis for Ecology. R package version 1.8‐0.

[ece33123-bib-0043] Shehzad, W. , McCarthy, T. M. , Pompanon, F. , Purevjav, L. , Coissac, E. , Riaz, T. , & Taberlet, P. (2012). Prey preference of snow leopard (*Panthera uncia*) in South Gobi, Mongolia. PLoS One, 7(2): e32104. 22393381 10.1371/journal.pone.0032104 PMC3290533

[ece33123-bib-0044] Shehzad, W. , Riaz, T. , Nawaz, M. A. , Miquel, C. , Poillot, C. , Shah, S. A. , … Taberlet, P. (2012). Carnivore diet analysis based on next‐generation sequencing: Application to the leopard cat (*Prionailurus bengalensis*) in Pakistan. Molecular Ecology, 21, 1951–1965. 22250784 10.1111/j.1365-294X.2011.05424.x

[ece33123-bib-0045] Taylor, P. G. (1996). Reproducibility of ancient DNA sequences from extinct Pleistocene fauna. Molecular Biology and Evolution, 13, 283–285. 8583902 10.1093/oxfordjournals.molbev.a025566

[ece33123-bib-0046] Thomas, A. C. , Jarman, S. N. , Haman, K. H. , Trites, A. W. , & Deagle, B. E. (2014). Improving accuracy of DNA diet estimates using food tissue control materials and an evaluation of proxies for digestion bias. Molecular Ecology, 23, 3706–3718. 24102760 10.1111/mec.12523

